# Supplementary material for: Oral Squamous Cell Carcinoma Cells with Acquired Resistance to Erlotinib Are Sensitive to Anti-Cancer Effect of Quercetin via Pyruvate Kinase M2 (PKM2)
Source: Cells. 2023 Jan 1;12(1):179. doi: 10.3390/cells12010179 (PMC9818869; doi:10.3390/cells12010179)
Supplement: Supplementary file 1 [file cells-12-00179-s001.zip › cells-2049219-supplementary.pdf]

Supplementary Materials

A **B**

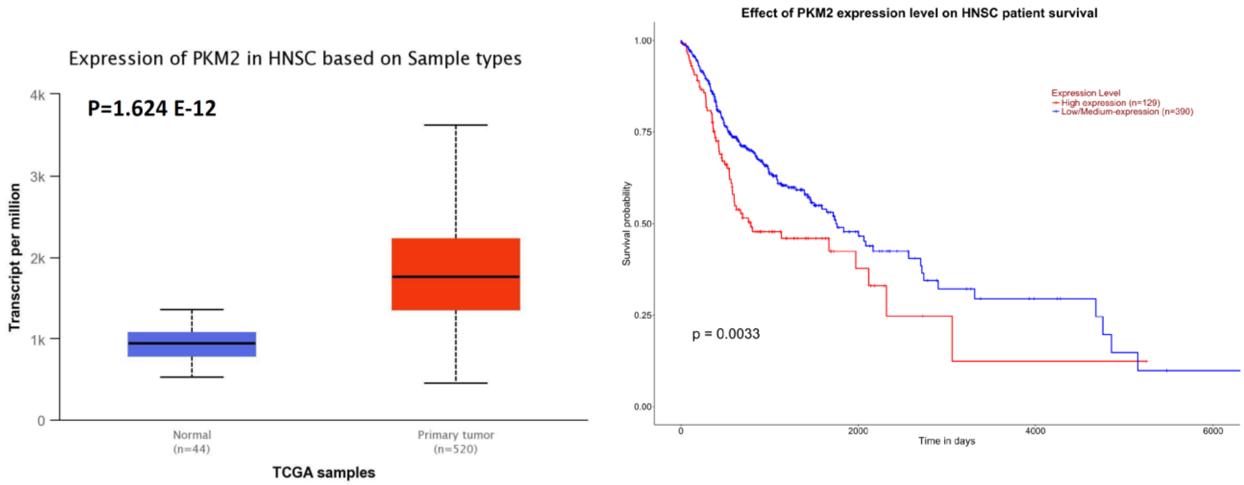

Figure S1. PKM2 expression in the HNSCC (Head and Neck squamous cell carcinoma) dataset of the Cancer Genome Atlas (TCGA) project. (A) Using the UALCAN (the University of ALabama at Birmingham CANcer data analysis) web portal, the mean expression of PKM2 in HNSCC primary tumors. (n=520) and e normal tissues (n=44) were analyzed. (B) Effect of PKM2 expression level on the HNSCC patient survival was analyzed by the Kaplan-Meier survival plotter. Results were considered significantly different at $p < 0.05$.

A **B**

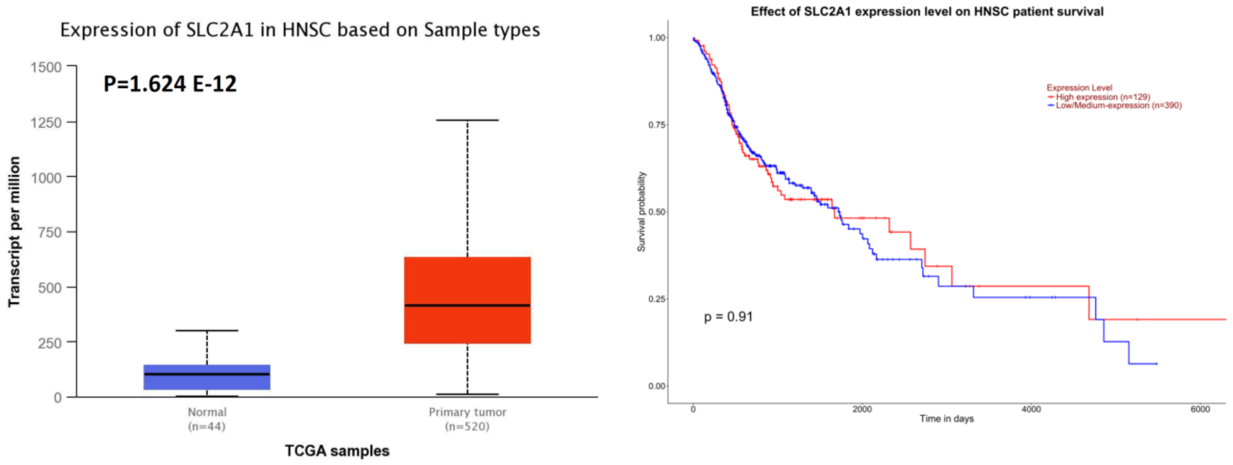

Figure S2. GLUT1 (SLC2A1) expression in the HNSCC (Head and Neck squamous cell carcinoma) dataset of the Cancer Genome Atlas (TCGA) project. (A) Using the UALCAN (the University of ALabama at Birmingham CANcer data analysis) web portal, the mean expression of PKM2 in HNSCC primary tumors. (n=520) and e normal tissues (n=44) were analyzed. (B) Effect of GLUT1expression level on the HNSCC patient survival was analyzed by the Kaplan-Meier survival plotter. Results were considered significantly different at $p < 0.05$.

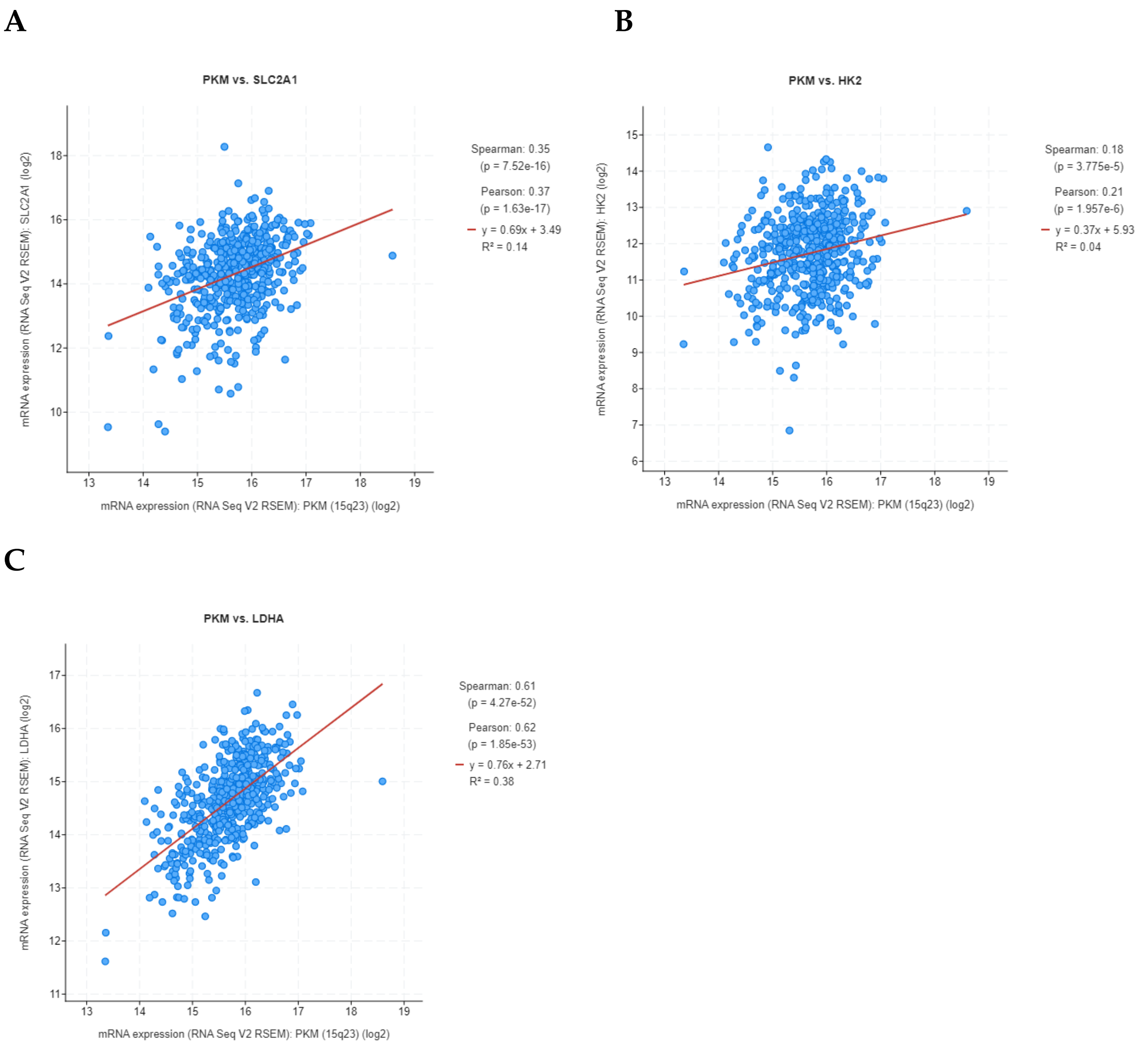

Figure S3. PKM2 mRNA expression positively correlates with glycolytic enzyme genes in an HNSCC (Head and Neck squamous cell carcinoma) dataset of the Cancer Genome Atlas (TCGA) project. From the cBioPortal for cancer genomics engine website, co-expression analysis of an HNSCC dataset (530 samples) showed positive correlation between PKM (PKM2) and (A) SLC2A1 (GLUT1); (B) HK2; (C) LDHA. The mRNA expression levels were expressed as log2, and the results were considered significantly different at $p < 0.05$ by Spearman and Pearson correlation test.

A

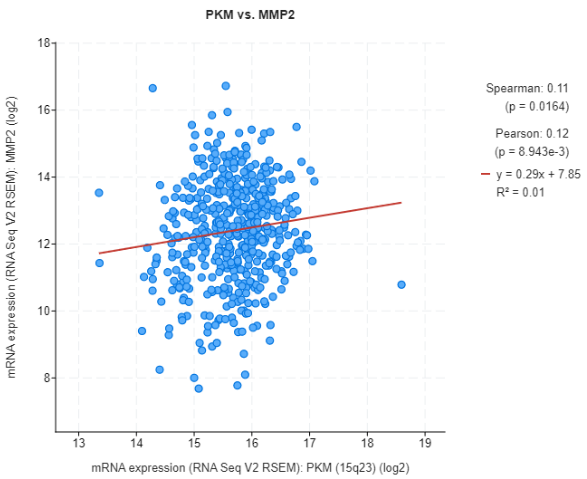

B

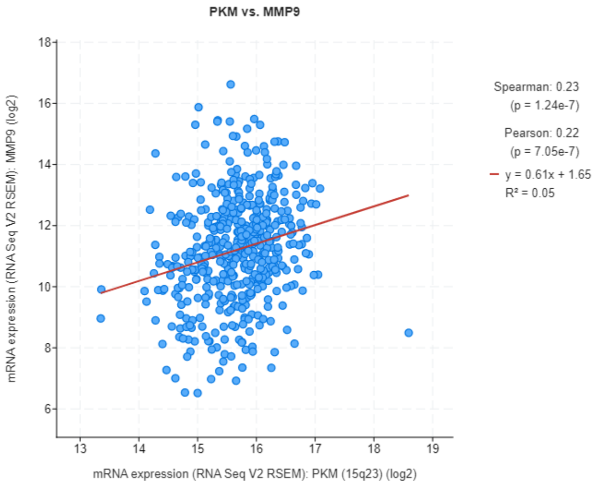

C

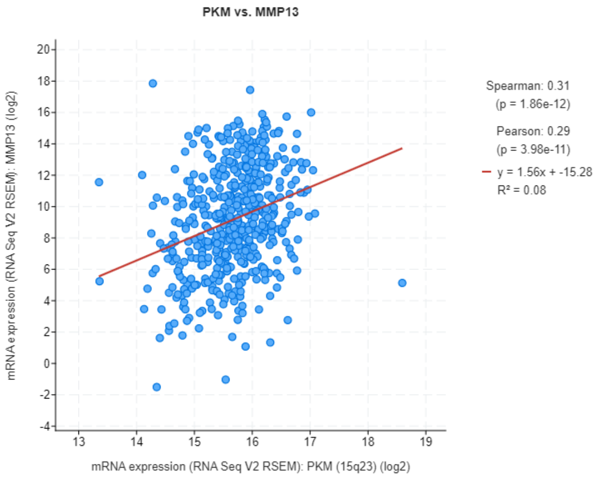

Figure S4. PKM2 mRNA expression positively correlates with matrix metalloproteinase (MMP) genes in an HNSCC (Head and Neck squamous cell carcinoma) dataset of the Cancer Genome Atlas (TCGA) project. From the cBioPortal for cancer genomics engine website, co-expression analysis of an HNSCC dataset (530 samples) showed positive correlation between PKM (PKM2) and (A) MMP-2; (B) MMP-9; (C) MMP-13. The mRNA expression levels were expressed as log2, and the results were considered significantly different at $p < 0.05$ by Spearman and Pearson correlation test.
